# Suitability of early *indica* rice for the preparation of rice noodles by its starch properties analysis

**DOI:** 10.1016/j.fochx.2024.101921

**Published:** 2024-10-23

**Authors:** Saadia Zainab, Xianqing Zhou, Yurong Zhang, Saira Tanweer, Tariq Mehmood

**Affiliations:** a College of Food Science and Engineering, Henan University of Technology, Zhengzhou, China; b Department of Food Science and Technology, Faculty of Agriculture and Environment, The Islamia University of Bahawalpur, Pakistan; c Institute of Food Science and Technology, Faculty of Food, Health Science and Technology, Khwaja Fareed University of Engineering and Information Technology, Rahim Yar Khan, Pakistan

**Keywords:** Early *indica* rice, Rice noodles, Starch properties, Starch structure, Quality analysis

## Abstract

The research was conducted to explore the affiliation between the physicochemical properties of rice noodles and rice starch of early *indica* rice samples of varying amylose content. 3 various types of rice samples were analyzed to uncover how amylose content influences rice noodles' quality. Findings revealed that primarily sensory scores differ in palatability, while textural disparity lies in hardness and chewiness. The higher hardness and chewiness values were correlated with higher sensory scores. Rice with lower amylose content demonstrates elevated solubility, swelling power, and crystallinity along with poor retrogradation resulting in inferior-quality noodles. Sensory scores and textural properties were proved to be associated with the distribution of branched starch molecule chain lengths. Noodles of higher sensory scores had more stable gel structure and improved elasticity. These findings underscore the critical role of amylose content and starch molecular structure in determining the suitability of early *indica* rice for noodle preparation.

## Highlights

• Amylose content significantly affects the eating quality of rice noodles
• Higher amylose content improves the sensory and texture of rice noodles
• Lower crystallinity, solubility, and swelling power enhance the eating quality of rice noodles
• Amylopectin's higher hydrolysis rate contributes positively to the properties of rice noodles

## 1 Introduction

Rice noodle is a traditional food with a long tremendous history. It is a kind of strip or linear rice product made from rice through a series of processing techniques. Rice noodles are divided into pressed noodles (mostly round-type noodles) and cutting flat noodles (flat-type noodles) according to the different production processes and appearance shapes. Noodles have attracted quite a lot attraction of people in recent years because of their high digestibility and hypoallergenic characteristics. Some rice noodles in China are manufactured by using the conventional steamed approach in which noodles are shaped into strips also known as flat rice noodles (Jiang et al., 2024). Because of its distinctive texture, flavor, and predilection, rice noodles are quite popular among many Southeast and East Asian inhabitants. Rice noodles are getting attention due to increase in gluten-free diets. Noodles have become a staple food for those seeking such a diet. Noodles are considered as best substitute used for celiac disease patients because of the deficiency of gluten proteins which is an autoimmune chronic disorder caused by dietary gluten usually found in barley, rye, and wheat (Chen et al., 2023; Chen et al., 2024). Besides nutritional facts, the global market in 2020 was recorded at USD 4.73 and it is predicted to spread up to 9.51 by 2027 and for 2030, it is likely to be touched USD 12.65. From the market analysis of 2021, it is evident that the consumption mandate for instant noodles has inflated progressively in recent years (Kraithong et al., 2023). Which instigate the researchers to focus more on noodles to explore new insights.

Rice noodles' acceptance can be linked to their pliable texture and smooth taste. Noodles are mostly and widely consumed in China and they exist in extensive categories and forms like white salt noodles, udon noodles, ramen, and raw and wet noodles (Yan et al., 2023). Not only this, cooked noodles are available in a wide range with diverse quality attributes. Based on moisture content, rice noodles can be divided into dried, semi-dried, or packaged noodles. Fresh noodles are preferred by customers over dried noodles owing to their freshness, time-saving, and expedient use. On the other hand, dried noodles took a long time to cook. However, fresh noodles' shelf life is comparatively limited because of the possibility of spoilage by bacteria due to a high moisture content of more than 50 % (Guo et al., 2023). Rice noodles are conventionally prepared with high-amylose rice flour (more than 25 %) or from mediocre-amylose rice flour (20–25 %) (Kraithong et al., 2023).

*Indica*-type rice with high AC is usually used for manufacturing rice noodles, while Japonica rice may be mixed partially to adjust the noodle texture (Lu et al., 2009). *Indica* rice is preferred for rice noodle production due to its good shininess, flavor, and higher springiness, which contribute to better-quality noodles. AC is considered one of the key detrimental factors for the quality evaluation of rice noodles as it plays a crucial role in forming the starch network (Wei et al., 2022). Starch is the main constituent of rice grain (≈90 % dry basis) influencing almost every core property of rice, so its characteristics play a vital role in ascertaining the quality of rice noodles (Yi et al., 2020).

To best of our knowledge, there are not so many studies carried out to highlight the role of starch in improving the rice noodle eating qualities by focusing on both AC and AP. So far, studies have been focused on the improvement of mechanical and cooking properties of rice starch (Jia et al., 2022), effect of starch structure on the digestibility, texture, and flavor quality of rice noodles (Shen et al., 2021), compositional, thermal, and rheological influence on rice noodles (Wei et al., 2022), the effect of rice proteins on the starch-lipid network in rice noodles (Chen et al., 2023), structural changes during retrogradation of rice noodles (Chen et al., 2024), and estimation of optimum cooking time of rice noodles (Guo et al., 2023). Little is known, however, the chain length distributions for each study previously conducted is determined through advanced techniques.

In this research, rice flour samples along with rice starch samples were analyzed to check their impact on noodles eating qualities. This study makes major contributions to the said theme. Firstly, this study is not only fixated on the AC but AP was also discussed along with the IRF and IRS samples to attain the noodles properties based on consumers' perspectives. Secondly, in this study for the determination of chain length distributions, chemical approach is used instead of some recent and advance method to highlight the chemical bonding and their role in determining the eating quality of rice noodles.

## 2 Methodology

### 2.1 Procurement of samples

Early *indica* rice samples, namely Jinzao-239, Zhejiang-1702, and Zhongzao-39, were procured from the Jinhua Academy of Agricultural Sciences, and their basic information is shown in Table S1 (Supplementary Data). The verities were selected based on AC, in which one variety has comparatively lower AC while the other two have minute differences in values so that the significance of AC role can be investigated. All reagents used were procured from Shanghai Yuanye Biotechnology Co., Ltd. Each rice sample was cleaned, hulled and milled. The polished rice samples were sealed in the zip lock bags for further use.

### 2.2 Determination of physicochemical properties

For the determination of physicochemical properties, AOAC and AACC international methods were used. To determine the moisture content AACC method 44–19.01, ash 08–01.01, starch 76–13.01, and amylose content 61–03.01 were used accordingly. AOAC methods 984.13 and 920.39 were used for the assessment of crude protein and crude fat respectively.

### 2.3 Rice noodle making

For the preparation of noodles, Shen et al. (2021) method was followed with minute alterations. MFD15 multi-functional 1-step molding machine was used for the preparation of the noodles as it is a fast approach ideally used because of lower energy consumption, visual quality, hygiene, compact, flexible regardless of low volume or mass production. 400 g of polished rice were weighed, washed 3 times with distilled water, and soaked in 1 L distilled water for 8 h. The water was collected after soaking for later use. 70 °C of distilled water was added slowly to the feed port of the 1-step molding machine followed by the addition of 50 g of rice for the test machine by reaching the standby temperature up to 45 °C and wait the stabilization of powder to introduce rest of rice to feed hopper along with the distilled water by maintaining the distilled water level less than 1 cm than the rice sample through extrusion process. The extruded rice noodles were placed on a stainless-steel rail for aging in a constant temperature and humidity chamber (15 °C and 70 %) for 6 h and stored in a zip lock bag for further use.

### 2.4 Textural characterization

For the determination of textural properties, 20 noodles strips of 20 cm length were taken. In a stainless-steel pot of diameter 22 cm, 500 mL of distilled water was added and brought to a boil. Rice noodles were placed in boiling water for 3 min and slight boiling was provided by controlling the induction cooker power. Noodles were removed and cooled for 2 min followed by cutting into the segments of 5 cm for the determination of textural properties (Guo et al., 2023). Parameters used for the TPA analysis were: test speed: 2 mm/s before the test, 1 mm/s during the test, 2 mm/s after the test, compression ratio 50 %, interval 5 s, induction force 5 g, parallel measurement 5 times (Chen et al., 2021).

### 2.5 Sensory evaluation of rice noodles

For the sensory evaluation, 20 cm long 20 noodle strips were selected after cooking after resting for 5 min. The methods were slightly modified and then adopted for the sensory assessment (Wei et al., 2022). The sensory evaluation was conducted at the standard sensory laboratory in the College of Food Science and Engineering, Henan University of Technology, Zhengzhou, China. 20 panelists including teachers and students with an equal ratio of male and female were selected on the basis of preliminary knowledge of sensory evaluation and their discrimination ability to differentiate various samples through their sensory sensitives by connecting them to sensory attributes. Volunteered participants signed the informed consent form to use the results anonymously. No additive or health risk element was involved in this study so no ethical consent was required.

Panelists were trained by following the ISO standards (ISO 8586:2012) for participation in sensory evaluation. Panelist must complete their organoleptic assessment within 10 min and must rinse their mouths with water between samples. The parameters assessed were odor (scent), appearance (color, lust, structural integrity, and uniformity), palatability (viscosity, hardness, sense of tendon), taste (flavor), and average score. The samples were assessed using a 100-point scale and the scores assigned to each characteristic were odor (0–15), appearance (0–25), palatability (0–35), and taste (0–25). The rules set for sensory scoring the rice noodles are explained in Table S2.

### 2.6 Extraction of *indica* rice starch

For the separation of rice starch, Jiang et al. (2024) method was adopted with slight modifications. 30 g of rice flour was passed through a 100-mesh sieve size that was added in a 500 mL beaker in conjunction with 150 mL of 0.02 mol/L sodium hydroxide solution and placed at room temperature for 3 h by providing magnetic stirring, followed by centrifugation (4500 r/min) for 15 min to remove the supernatant (yellowish layer). Centrifugation was done thrice with 100 mL of deionized water by making sure to remove the upper serum layer completely. pH of the solution was adjusted to neutral by using 1 mol/L HCl washed thrice with water and dried at 40 °C for 10 h. Fat was removed through the Soxhlet extraction method (refluxed for 8 h) and dried at the same conditions to acquire the samples that were grounded, sieved (100 mesh size), and refrigerated at 4 °C for later use.

### 2.7 Determination of gelatinization characteristics of IRF & IRS

Gelatinization characteristics of the rice flours and starches were determined through RVA (Perten Instruments, Warriewood, Australia) by following the method of Yang et al. (2021) after slight modifications.

### 2.8 Determination of solubility and swelling power of IRS

Wang et al. (2018) and Yi et al. (2020) approaches were followed with minute alterations for the determination of solubility and swelling power respectively.

### 2.9 Thermal Properties of IRS

For the assessment of rice starch samples DSC (Shimadzu, Japan) was used by following the method of Xiang et al. (2023) with slight amendments.

### 2.10 Crystal structure analysis of IRS

An XRD (D8 Advance, Bruker, Germany) was used to analyze the dried rice starch samples and a solid powder tableting approach was employed to acquire the X-ray diffraction spectrum of the sample. The parameters and method used for the crystallinity calculation were adopted by following the Bai et al. (2024) approach. The parameters set were: tube pressure 40 kV, tube current 40 mA, scanning speed 10°/min, diffraction angle range 5, emission angle 50°, step size 0.02°.

### 2.11 Fine structure analysis of amylopectin

Fine structure analysis of amylopectin can be further categorized into three steps that are explained in detail below:

#### 2.11.1 Isolation and purification of amylopectin

For the isolation and purification of amylopectin, the Mokhtari et al. (2024) approach was adopted with slight alterations. After defatting and deproteinization, 1 g of rice starch was weighed in a 50 mL centrifuge tube in which 2 mL of absolute ethanol along with 30 mL of 0.5 mol/L sodium hydroxide solution was added to disperse the sample and heated in a water bath by providing the stirring until clear, transparent and agglomeration free solution acquired. The solution was cooled at room temperature and centrifuged at 8000 r/min for 20 min to remove the impurities. After centrifugation, the solution was neutralized with 2 mol/L HCl and kept for 24 h at 4 °C followed by centrifugation (8000 r/min, 20 min, 4i). The final centrifuged solution acquired by discarding precipitates was crude amylopectin which was allowed to stand in a separating funnel for 30 min. The lower layer of latex-like solution was accumulated in which 5 mL of n-butanol-isoamyl alcohol (volume ratio 1:1) was incorporated and heated with a stirrer assisted for 20 min to get the clear and transparent solution subsequent to cooling at room temperature. The solution was again placed in the refrigerator at 4 °C for 24 h. The centrifugation was again done at a lower temperature 4 °C, by providing the conditions of 8000 r/min and 20 min. The precipitate was discarded and the supernatant was again undergone through centrifuge process 4 times. At the end of the process, 2 times of volume of cold absolute ethanol was added and placed for 6 h at 4 °C to precipitate the starch which was subsequently subject to centrifugation (5000 r/min and 10 min). The precipitate obtained was washed with absolute ethanol 3 times which was dried for 10 h at 4 °C to get the purified amylopectin. The flow line for the isolation and purification of amylopectin is depicted in Fig. S1.

#### 2.11.2 Determination of the hydrolysis rate of amylase β

Gui et al. (2021) method with slight modifications was adopted for the assessment of the hydrolysis rate of β-amylase. 50 mg of purified amylopectin was taken in a centrifuge tube in which 2.5 mL of acetic acid buffer (0.02 mol/L, pH 5.0) was introduced and mixed well. 5 mg of β-amylase (250 U) was also appended and heated at 37 °C for 48 h. The reaction solution was kept in a boiling bath for 20 min to stop the reaction. The solution was cooled at room temperature. Phenol sulfuric acid method and dinitrosalicylic acid method (DNS) were used for the approximation of total sugars (S) and total reducing power (W, calculated as maltose). For the calculation of the hydrolysis rate of β-amylase (D, %), the following formula was used.

$$\beta -\mathrm{amylase hydrolysis rate}\;\left(\%\right)=\frac{\mathrm{Reducing power}}{\mathrm{Total sugars}}\times 100$$

#### 2.11.3 Determination of amylopectin chain length structure

The method adopted for the estimation of amylopectin chain length structure was explained by Hu et al. (2020). 22 mg of pure amylopectin was taken in 5 mL of centrifuge tube in addition to acetic acid buffer (0.05 mol/L, pH 5.0) and 4 mg of pullulanase (4u), mixed well and heated at 37 °C for 24 h. After the cooling at room temperature, total sugars (S) and total reducing power (W, calculated as glucose) were determined through the phenol sulfuric acid approach and dinitrosalicylic acid approach respectively. For the valuation of average chain length (CL), outer chain length (ECL), and inner chain length (ICL), the following formulas were used.

$$\mathrm{CL}=\frac{\mathrm{Total carbohydrates}\;\left(\mathrm{Glucose equivalent}\right)}{\mathrm{Total reducing sugar}\;\left(\mathrm{Glucose equivalent}\right)}$$

ECL=CLxβ−amylase hydrolysis rate+2.0

$$\mathrm{ICL}=\left(\mathrm{CL}-\mathrm{ECL}\right)-1.0$$

### 2.12 Determination of retrogradation of IRS

After minor alterations, the method was employed. 0.25 g of dry-based starch was weighed to prepare 1 % starch suspension and heated in the water bath for 30 min, vortexed, and mixed every 5 min by keeping the total volume of the sample the same throughout the process. The solution was cooled at room temperature and shifted into a 50 mL calibrated cylinder. Readings of supernatant volume and total volume were noticed after every 12 h. The retrogradation was calculated according to the formula (L. Wang, Wang, et al., 2022).

$$\mathrm{Retrogradation}\;\left(\%\right)=\frac{\mathrm{Volume of supernatant}\;}{\mathrm{Total Volume}}\times 100$$

### 2.13 Rheological properties of IRS gels

After the assessment of gelatinization properties by RVA, samples were cooled down at room temperature and later subjected to a rheometer for the determination of rheological properties. The static rheological parameters set at: shear mode, 40 mm plate carrier, plate spacing 1 mm, and shear range of 0.1 to 150 s^−1^ while dynamic rheological parameters adopted as: temperature 25 °C, strain 1 % and frequency range of 0.1 to 10 Hz (Yan et al., 2023). The G' and G" curves are fitted to the power-law model, and the coefficient of determination R^2^ represents the fitting accuracy of the equation.

$$G^{\prime }=K\prime \omega^{n\prime }$$

$$G^{\prime \prime }=K^{\prime }\prime \omega^{n\prime \prime }$$

where K′ and K″ are constants, ω is the angular frequency (rad/s), and n’ and n” are the frequency indexes.

From the values of storage and loss modulus, the complex modulus was calculated by using the following formula.

$$G*=\surd {\left(G^{\prime }\right)}^{2}+{\left(G^{\prime \prime }\right)}^{2}$$

### 2.14 Data statistics and analysis

Experiments were conducted in triplicates and data was represented as mean ± standard deviation (SD). Data was organized and plotted using Excel 2019 and origin2018. IBM SPSS Statistics 2023 (SPSS Inc. Chicago, IL, USA) was used for statistical analysis. Variance analysis was conducted through Duncan's multiple comparison method for significance testing and *p* < 0.05 was considered a significant difference.

## 3 Results and discussions

### 3.1 Physicochemical and Gelatinization properties of rice

#### 3.1.1 Physicochemical properties of rice

Chemical and physical characteristics play a vital role in determining the rice quality (Hu et al., 2020). The structural characteristics of rice noodles are mainly influenced by the starch, lipids, and proteins. The physicochemical properties of the three types of rice are shown in Table 1. AC is the main detrimental factor in the assessment of the quality of noodles. Noodles prepared from the rice of AC (20–25 %) are considered best in quality. Higher AC improved the hardness of noodles and sensory quality while decreasing breakage rate and cooking loss (Kraithong et al., 2023). 3 had the highest value of AC after the 2 and 1 accordingly representing that noodles prepared from 3 had good quality parameters. Starch and protein networks play a key role in the gelatinization and retrogradation properties as both during heating compete for the water and disturb the swelling and rupturing of starch granules (Xu et al., 2019). 3 had the lowest protein value followed by samples 2 and 1. Although there is a slight difference in protein value among samples that had a huge impact on pasting parameters.

**Table 1:** Physicochemical (%) and gelatinization parameters of *Indica* rice samples.

Physicochemical properties						
Samples	Moisture	Ash	Fat	Protein	Starch	Amylose
1	10.92 ± 0.00b	0.59 ± 0.00a	1.08 ± 0.07a	7.87 ± 0.03a	78.67 ± 0.33a	16.29 ± 0.16a
2	11.45 ± 0.05a	0.38 ± 0.01b	0.63 ± 0.07b	7.28 ± 0.03b	75.79 ± 0.38b	24.36 ± 0.13b
3	10.42 ± 0.01c	0.32 ± 0.00c	0.32 ± 0.01c	7.27 ± 0.04b	75.54 ± 0.87b	25.43 ± 0.05c


Gelatinization properties						
Samples	PV (cP)	TV (cP)	BV (cP)	FV (cP)	SV (cP)	GT (°C)
1	1540.00 ± 18.38a	1288.50 ± 2.12a	251.50 ± 16.26a	2104.00 ± 14.14a	815.50 ± 12.02a	83.10 ± 0.34a
2	3831.00 ± 48.08b	2977.00 ± 9.90b	854.00 ± 57.98b	4621.00 ± 70.71b	1644.00 ± 80.61b	81.20 ± 0.27b
3	3841.50 ± 48.79b	2828.50 ± 10.61c	1013.00 ± 38.18c	4488.00 ± 28.28b	1659.50 ± 3.54b	81.55 ± 0.23b

Different letters in the same column indicate significant differences (*P* < 0.05).

Starch is the main constituent having 75 % of the total rice composition while the remaining parameters altogether score up to 25 %. 1 had the highest starch value and the lowest AC as compared to the other two rice samples. Additionally, there were significant differences among moisture, ash, and fat content of the three *indica* rice samples. The variation in the physicochemical values can also be attributed to the variety, environment, agronomic, and other factors (Kakar et al., 2020).

#### 3.1.2 Gelatinization properties of rice

The gelatinization process of starch represents water absorption, swelling, destruction of crystal structure, and amylose leaching (Cheng et al., 2024). The water absorption capacity of starch tremendously affects the texture and taste of rice noodles. Relatively higher water absorption can improve the softness and palatability of noodles while lower absorption can result in a firmer and drier texture (Wei et al., 2022). RVA pattern and values of gelatinization parameters of IRF are depicted in Fig. S2 (A) & Table 1.

Sample 2 and 3 have almost the same amplitude of curves, while 1 had a smaller one. 1 had the higher gelatinization parameters' values in comparison to other samples which can be associated with the amount of amylose content. The gelatinization parameters' values followed the trend 3 > 2 > 1 which aligns with the research conducted by Wei et al. (2022). Among all, 1 had more protein content and starch values. Starch and protein formed a complex network for which more temperature is needed to break down the structure and higher viscosities values were recorded. Fat also plays a key role in determining the gelatinization properties. Fat exists on the surface while the starch granules encapsulated in the protein complex structure) make starch granules rupture after the water absorption and swelling (Xu et al., 2019). The retrogradation phenomenon is advantageous for the noodles as it helps in enhancing the textural and viscoelastic properties of the noodles (Jiang et al., 2024). By considering this fact 3 had more probability of undergoing smooth retrogradation phenomenon in comparison to the other two samples.

### 3.2 Eating quality and texture profiles of rice noodles

**Fig. 1:**
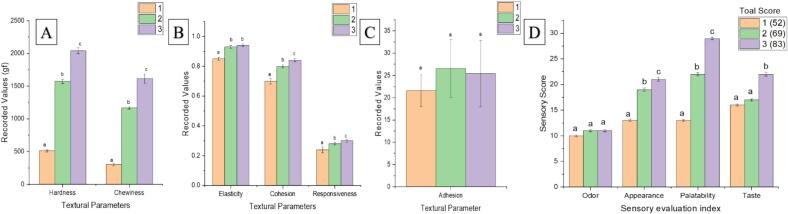
Textural characteristics & Sensory evaluation score of noodles.

Sample 3 (Fig. 1D) had the highest sensory comprehensive score (83 points), and 1 had the lowest sensory comprehensive score (52 points). The difference in sensory score is mainly in the palatability and appearance with minute variations in smell. Higher appearance score reflects noodles with more luster, off-white color, compact structure with no cracks, and uniform smoothness and thickness. Lu et al. (2022) explained in their research that noodles with low AC were found to have a softer texture even though having an increased protein content. Palatability is the joint response of stickiness, hardness and softness, and sense of tendon. Improved palatability indicates non-sticky, moderate hardness, and chewy characteristics of the noodles while a higher taste score reflects strong rice flavor (Kraithong & Rawdkuen, 2021).

Texture characteristics showed that there were no significant differences in hardness, chewiness, cohesion, and recovery. The major recorded differences were between hardness and chewiness. Hardness and chewiness values of noodles prepared by 1 rice flour were significantly lower than those of 2 and 3. Wei et al. (2022) also got the same results regarding noodles' texture and sensory by explaining the significance of hardness and chewiness in affecting the taste of rice noodles. AC plays a major role in the quality of rice noodles, and the sensory quality of rice flour produced by processing *indica* rice with AC of 21 % ∼ 25 % is better (Kraithong et al., 2023). AC is strongly correlated to the textural properties of the noodles. A strong construction by 3D network is formed by amylose which is supported by the disulfide bonds of amino acids from protein. Generally, elevated values of hardness, elasticity, and cohesiveness represent good-quality rice noodles (Kraithong & Rawdkuen, 2021). The AC of 1 is 16.29 % which is the lowest among the three rice samples, so the rice noodles produced from them are of the worst quality among the three types. Among 2 and 3, the sensory comprehensive score of rice flour prepared from 3 was higher, which may be related to the starch structure, as starch being the main component of rice, is the main factor affecting the edible quality of rice flour, so the analysis of starch characteristics helps analyze the reasons for the difference in the eating quality of rice noodles.

### 3.3 Physicochemical, gelatinization, and thermal properties of rice starches

#### 3.3.1 Solubility & swelling power and retrogradation property

**Fig. 2:**
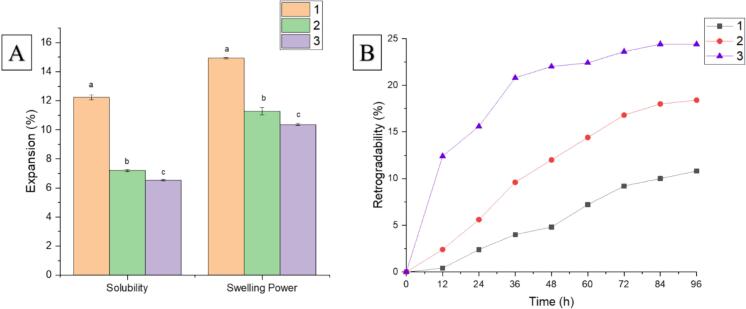
Solubility and swelling power (A) and degree of retrogradation (B) of IRS.

Significant differences were recorded in the solubility and swelling power (Fig. 2A), among which 1 had the highest solubility and swelling power, while a slight difference was recorded between 2 and 3. The difference can be related to the content of amylose as its tight helical structure inhibits the expansion of starch granules (Zhong et al., 2022). The same trend was obtained by Yi et al. (2020). Solubility and swelling power results are consistent with the breakdown and peak viscosity values of rice starches. This can be linked to the leaching of AC during the starch gelatinization due to the alterations in hydrogen bonding that occurred. Additionally, the lipid-amylose complex lowered the charged molecules and restrained the swelling of rice flour (Farooq et al., 2021). Lower swelling power revealed comparatively less absorption of water, leading to medium noodles while reduced solubility pointed to the less starch leaching and retention of denser and potentially chewier texture (Jiao et al., 2020) that align with texture profile results.

It can be seen from Fig. 2B that within 0–12 h, the starch of the three samples immediately turned into illegible coagulation and the coagulation increased steadily in a straight line. Among all, 3 had the largest value. At the 12th hour of the experiment, the rate of 3, 2, & 1 reached 12.4, 2.4, and 0.4 % accordingly, exhibiting 3 had the maximum ability to form hydrogen bonds among molecule chains that easily aggregate. During 12–84 h, the rate of three samples gradually increased and after 84 h there was no significant difference in values obtained at the 96th hour value that can be attributed to the sedimentation process over a long time.

Throughout the experiment, the increment trend of values obtained was 3 > 2 > 1 indicating that starch in 3 had more contribution in forming dense, stronger, and harder noodle structures as compared to the other ones. Leaching of amylose after gelatinization contributes to the formation of a double helix in the gel in the cooling process. This winding and orderly organization of amylose with the combination of gelatinization results in enhanced gel strength (Chakraborty et al., 2024). Rice starch with a high degree of retrogradation participated in the preparation of more delicate, consistent, tasty, ductile, and elastic noodles (Jia et al., 2022). So, 3 had a higher value of AC and degree of retrogradation, which contributed to making rice noodles tasty and smooth.

#### 3.3.2 Gelatinization properties of rice starches

In comparison to IRF, IRS samples have higher values of setback and decline in gelatinization time and a minute difference in gelatinization temperature was observed that can be associated with the constitutional components like proteins, and fats that can affect the gelatinization process of rice grains resulting in differences in rice flour and rice starch values. Defatting and deproteinization processes result in the reduction of peak time. The shorter the peak time of rice starch, the more viscosity increases making it easy to rapidly age and decline the thermal stability (Yang et al., 2021). It can be seen from Fig. S2 (B) & Table 2 that among the three types of rice starch, 1 has higher peak viscosity and breakdown value and lower setback value, indicating that the thermal stability of 1 starch paste is poor and the strength of the aging gel is weak. This is consistent with the linear chain of amylose, the content of starch, and the hydrogen bonds cross-link and polymerize starch molecular chains together. A high value of AC and high hydrogen bonds improve the hardness and elasticity of gel formed that directly influenced the hardness, viscoelasticity (Cheng et al., 2024), and other textural properties of rice noodles (Wang et al., 2024). 2 and 3 have almost the same AC but 3 has a lower breakdown value and higher setback value. The breakdown value depicts the stability of starch hot paste i.e., resistance to the shear and heat (Lin et al., 2023) while the setback represents the stability and aging tendency of the starch cold paste i.e., retrogradation. The same trend was achieved by Mauro et al. (2023) which can be because of high amylose content that leached during heating and blocked the granule swelling resulting in reduction of peak viscosity. Sample 3 is more resilient to the shear resistance, easy to age, and forms a strong gel. Rice noodles prepared from 3 are more appropriate in comparison with the other two as they have better elasticity and taste, and are hard to deform.

**Table 2:** Gelatinization and power law model parameters of IRS.

Gelatinization properties						
Samples	PV (cP)	TV (cP)	BV (cP)	FV (cP)	SV (cP)	GT (°C)
1	3428.00 ± 32.53a	1719.00 ± 8.49a	1709 ± 24.04a	3117.00 ± 43.84a	1398.00 ± 35.36a	84.15 ± 0.28a
2	3091.00 ± 134.35b	2102.00 ± 21.21b	989 ± 113.14b	3717.50 ± 3.54b	1615.50 ± 24.75b	80.63 ± 0.32c
3	3060.00 ± 12.73b	2087.00 ± 22.63b	973 ± 35.36b	3920.00 ± 35.36c	1833.00 ± 12.73c	82.23 ± 0.25b


Power function model parameters						
Samples	G'			G"		
	K_1_	n_1_	R^2^	K_2_	n_2_	R^2^
1	203.435	0.135	0.999	44.008	0.258	0.997
2	452.436	0.081	0.984	33.673	0.229	0.935
3	564.51	0.076	0.962	33.89	0.299	0.997

Different letters in the same column indicate significant differences (P < 0.05).

#### 3.3.3 Thermal properties of rice starches

DSC allows accurate determination of the heat flow associated with the starch gelatinization process (Farooq et al., 2021). The results obtained (Table 3) followed the trend 1 > 2 > 3 for almost all values which aligns with the values of gelatinization temperature acquired through RVA analysis. Lower crystallinity also results in lower gelatinization temperatures (onset, peak, and conclusion) and lesser enthalpy values as less energy is needed to disrupt the well-organized crystalline structure and cause the rapid melting of starch granules during the heating process (Li et al., 2022). DSC parameters are critical measures that directly influence the texture and quality of noodles. Higher values of To, Tp, and Tc indicate that more starch resistance towards gelatinization results in harder texture while a higher ΔH value depicts more energy requirement and breakdown of starch. Considering these sample 3 is the best fit for the noodle's preparation.

**Table 3:** Thermal properties of IRS.

Samples	To (°C)	Tp (°C)	Tc (°C)	ΔH (J/g)
1	70.56 ± 0.05b	84.78 ± 0.45a	91.25 ± 0.58a	13.70 ± 0.38a
2	74.70 ± 0.14a	81.98 ± 0.37b	88.17 ± 0.55ab	11.82 ± 0.36b
3	72.45 ± 0.07ab	80.24 ± 0.35b	86.93 ± 0.50b	10.56 ± 0.35b

Different letters in the same column indicate significant differences (P < 0.05).

### 3.4 Rheological characteristics of IRS gels

**Fig. 3:**
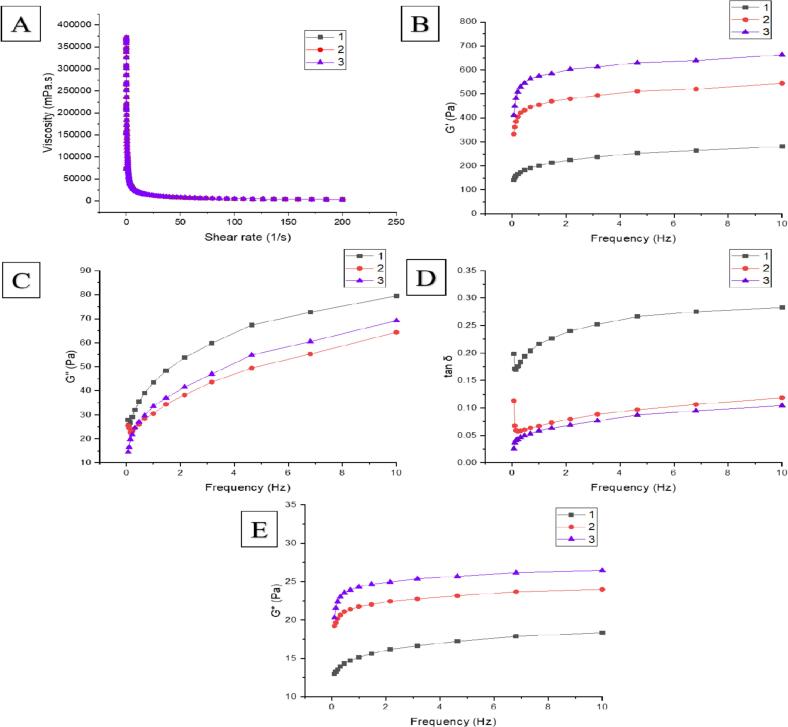
Rheological properties of IRS gels. A: Dynamic viscosity curves, B: Frequency sweep curves (G'), C: Frequency sweep curves (G"), D: loss tangent curves, E: Complex modulus curves (G*).

The decline in apparent viscosity of the three samples can be observed at a low shear rate, and then the values increased with the increase of shear rate (Fig. 3A). This type of trend belongs to the shear thinning phenomenon unique to pseudoplastic fluids, that is, the apparent viscosity of the fluid decreases with the increase of shear rate (Hassan et al., 2022). It is often used to express the shear stability of a system during the shear process. The curves of 2 and 3 inclined to coincide, but the apparent viscosity of 3 had the largest value, dominating among all, indicating that the sample 3 starch paste required greater shear force, high viscosity, and more stable gel structure at the same shear rate and the rice flour made was more elastic in taste. The higher proportion of amylopectin with greater polymerization could produce greater resistance to the flow, resulting in a decrease in the apparent viscosity of starch (Yan et al., 2021).

The frequency sweep mode is used to reflect the changes in the viscoelastic and network structure of the starch gel structure (Ramli et al., 2022). In dynamic rheological properties, Complex modulus G*, storage modulus G', and loss modulus G" reflect the strength, elasticity, and viscosity of the system, respectively. Tanδ, known as the loss tangent, is the ratio of viscous modulus to elastic modulus, the larger the value, the greater the viscosity of the fluid. Table 2 shows the power function model parameters of the simulated rheological curves, K_1_ and K_2_ are the consistency coefficients and viscosity of the reaction fluid, n_1_ and n_2_ are the fluid indices. The three samples (*n* < 1) indicate that all are pseudoplastic fluids, and the R^2^ is close to 1 indicating that the fitting degree between the theoretical fitting curve and the measured curve is high. The G', G", tanδ (G"/G'), and G* of the three samples always increase with the increase of scan frequency (Fig. 3B, C, D, E), where within samples the relationship of G', G* is 3 > 2 > 1, while G" is 1 > 3 > 2 samples G' which align with the results obtained by Wang, Chen, et al. (2022) which can be attributed higher AC as it contributes in more ordered and crystalline structure leading to greater elasticity and gel strength. Higher the G* values higher viscosity and strength will be needed to deform amplifying samples' good stability towards deformation.

The most variations in tanδ occurred in the range of 0.02–0.3 by exhibiting the trend of sample values 1 > 2 > 3 representing elastic behavior. 3 had the highest value that can be attributed to the AC, conducive to the formation of starch gel structure, and formed the gel system of greater rigidity and gel strength. Rice starch gels formed by high AC were found to be stronger and exhibit irreversibility (Mi et al., 2024).

### 3.5 Fine structure analysis of rice amylopectin

β-amylase acts on the α-1,4 glycosidic bond at the non-reducing end of the amylopectin molecule. It cannot hydrolyze the α-1,6 glycosidic bond that is responsible for branch points in amylopectin, nor can it continue hydrolysis across the branch point. Usually, the higher hydrolysis rate means that there are fewer remaining intact amylopectin molecules while the majorities have gone through the breaking process (Li et al., 2021). CL refers to the average number of glucose residues contained in the chain of the non-reducing end group of amylopectin, ICL is the chain length between the branch points of two adjacent α-1,6 glycosidic bonds, and ECL is the chain length from the outermost branch point of amylopectin to the non-reducing end, which forms a double helix structure through hydrogen bonding, and the longer the outer chain, the more stable the starch structure (Hu et al., 2020).

**Fig. 4:**
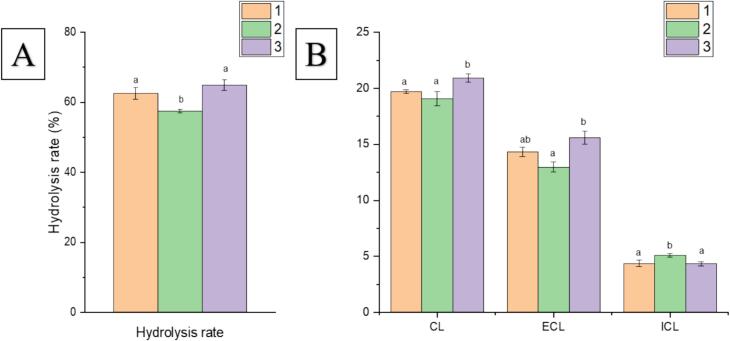
Chain length distributions of amylopectin.

The hydrolysis rate, CL, and ECL of β amylase of No. 3 were significantly higher than those of No. 2 (Fig. 4), indicating that 3 had more long branches, and the longer branched chain would lead to an increase in the molecular weight of starch molecules. Higher AC contributes to forming a more stable gel during heating and also affects the gelatinization properties of starch (Tian et al., 2023). As an important part of starch, the chain length distribution of amylopectin changes the gelatinization characteristics of starch by changing the structure of the crystals, which affects the taste of rice products (Shen et al., 2021). The differences in sensory scores and texture characteristics of rice flour prepared from 2 and 3 with similar AC was mainly due to chain length distributions of AP. High amylose with long-chain amylopectin can significantly influence other properties like swelling resistance and enhancing viscoelasticity (Qiao et al., 2024) which are in consistent with the outcomes of this study. The average chain length and average outer chain length were positively correlated with the gelatinization temperature, and negatively correlated with the peak viscosity and breakdown value (Mauro et al., 2023).

### 3.6 Crystalline structure of rice starches

**Fig. 5:**
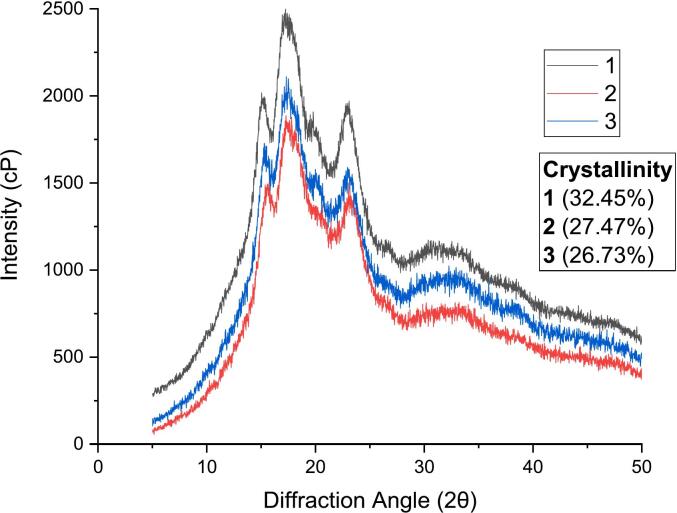
Crystalline structure of rice starch.

XRD curves are used to expose the statistics of the long-range ordered structure of rice noodles. Wider and diffused diffraction peak shapes can be referred to as weaker diffraction capability and insignificant crystallinity (Li et al., 2021). XRD patterns obtained were analyzed and peaks obtained at different 2θ with respective intensities are depicted in Fig. 5. Four peaks were obtained for each sample and the recorded 2θ values indicate A-type crystalline structure of rice starch (Table S3).

As can be seen from Fig. 5, the three kinds of rice starch have the characteristics of peak diffraction and diffuse diffraction at the same time, indicating that the three kinds of starch are composed of two parts, the crystalline and amorphous regions, which belong to the polycrystalline system, and the diffraction peaks are concentrated in 13° ∼ 25°. The obvious diffraction peaks can be observed near the diffraction angles of 15°, 17°, 18° and 23° with the diffraction angles of 15°, 18° and 23° have smaller diffraction peaks, and there are the strongest diffraction peaks near 17°, which belong to the typical A-type crystal structure. The main reflections of the XRD pattern at 2θ of 15°, 17°, 18°, and 23° exhibit A-type crystallinity (Gui et al., 2021) that is affiliated with the monoclinic crystal system mainly composed of a double helix structure molded by short side chains of amylopectin making the lattice structure of starch crystals more constricted. Crystallinity is significantly dependent on the chain length of amylopectin along with the great count of the short chain as they would contribute to crystallinity more. Although, too short chains of double helix, do not endorse the crystallinity of the sample (Cheng et al., 2022). The X-ray diffraction pattern of early *indica* rice 1 was high, indicating that the crystal had high crystallinity, high crystal structure integrity, and dense grain structure. Increment of AC can possibly disturb the crystalline regions formed by AP causing in reduction of crystallinity.

Starch crystallinity is an important indicator to measure the crystalline properties of starch, which refers to the percentage of the crystalline part of starch granules in the whole starch granules (Dome et al., 2020). The high crystallinity of 1 indicates that the crystalline area of no. 1 starch granules accounts for a large proportion, as compared to the crystallinity of 2 and 3 that were quite similar and the crystalline region of starch granules was formed by the aggregation of side chains of amylopectin molecules. The high crystallinity of starch granules makes it easier to form a uniform colloid and absorb more water during processing. The crystallinity of 1 is the highest, while the sensory quality of noodles prepared from 1 rice flour was the worst, which can be related to the structural distribution of amylopectin, and the consistency of the structure arrangement of amylopectin double helix that determines the crystallinity of starch (Dome et al., 2020). The content and distribution of amylopectin affect the crystallinity and morphology of starch; therefore, in the processing process of early *indica* rice flour, it is necessary to pay attention to control the content and distribution of amylopectin, to improve the taste and quality of rice flour. The amylopectin double helix structure in 1 starch sample is neatly arranged, the structure is compact, the crystallinity is high, and the temperature required for gelatinization is high, so it is not easy to make rice flour (Tian et al., 2023). Lower crystallinity leads to the improved gelatinization by enhancing water absorption capacity resulting in better texture as well as reduction in peak viscosity depicting the firm noodles (Wei et al., 2022).

## 4 Conclusion

The study was conducted to appraise the appropriateness of early *indica* rice noodles by focusing on the starch properties of three different rice samples. The experimental outcomes revealed significance differences in the sensory attributes and textural properties of rice noodles. Sample no. 1 with low AC exhibited lower values of hardness, chewiness, and overall sensory score making it inadequate for rice noodles production. Conversely, sample no. 2 and 3 having approximate same AC displayed notable differences in the sensory and textural properties. Sample no. 3, in particular, illustrated enhanced execution with highest sensory score, representing that this sample is most suitable among all for rice noodles preparation. The physicochemical properties and molecular structure analysis of rice starch strengthened the results obtained. Sample no. 3 had minimum breakdown value and maximum setback value among samples, exhibiting strong shear resistance and outstanding gelatinization characteristics. Lower crystallinity and longer amylopectin chain lengths also contribute to the superior quality of rice noodles. Assuredly, this research auspiciously recognized early *indica* rice sample no. 3 as most appropriate for rice noodles preparation on the basis of starch analysis and noodles eating quality and had achieved the research's objective. However, this research is based on small sample size so further research can be based on larger sample size to validate the obtained outcomes and to further explore the association between starch properties and rice noodles quality. Moreover, inclusion of processing factors and their impact on rice noodles quality would be valuable for the noodles production in further research and analysis.

## Use of AI tool

The authors declare they haven't used any AI tool.

## Funding

All the research experiments conducted were self-funded.

## CRediT authorship contribution statement

**Saadia Zainab:** Writing – review & editing, Writing – original draft, Funding acquisition, Conceptualization. **Xianqing Zhou:** Writing – review & editing, Supervision. **Yurong Zhang:** Resources, Conceptualization. **Saira Tanweer:** Writing – review & editing, Resources, Formal analysis. **Tariq Mehmood:** Visualization, Resources, Project administration.

## Declaration of competing interest

The authors declare that they have no conflict of interests.

## Data Availability

Data will be made available on request.

## Appendix A Supplementary data

Supplementary material Image 1
