# Supplementary material for: Suitability of early indica rice for the preparation of rice noodles by its starch properties analysis
Source: Food Chem X. 2024 Oct 23;24:101921. doi: 10.1016/j.fochx.2024.101921 (PMC11550054; doi:10.1016/j.fochx.2024.101921)
Supplement: Supplementary file 1 — Supplementary material [file mmc1.docx]

**Suitability of early *indica* rice for the preparation of rice noodles by its starch properties analysis**

**Supplementary Data**

**Figures Caption:**

Fig. S1. Flow line of (A) Isolation and (B) Purification of amylopectin

Fig. S2. Pasting parameters of (A) *Indica* rice flour and (B) *Indica* rice starch

**Figures:**

**
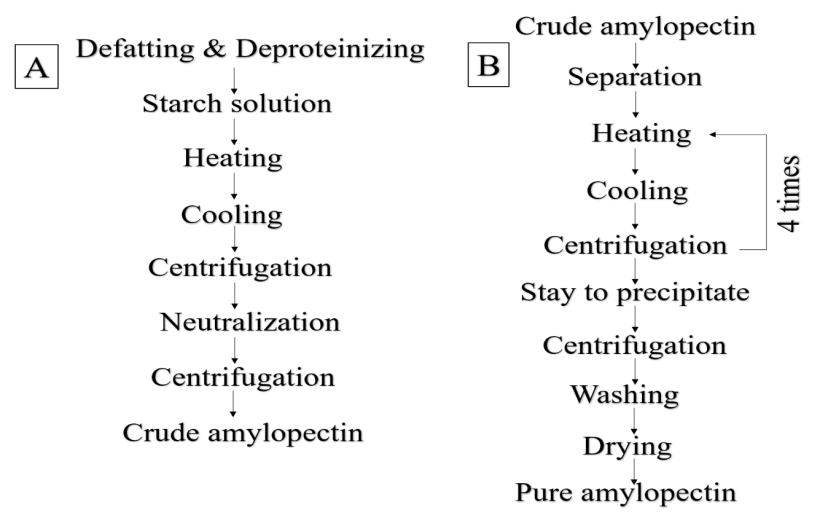
**


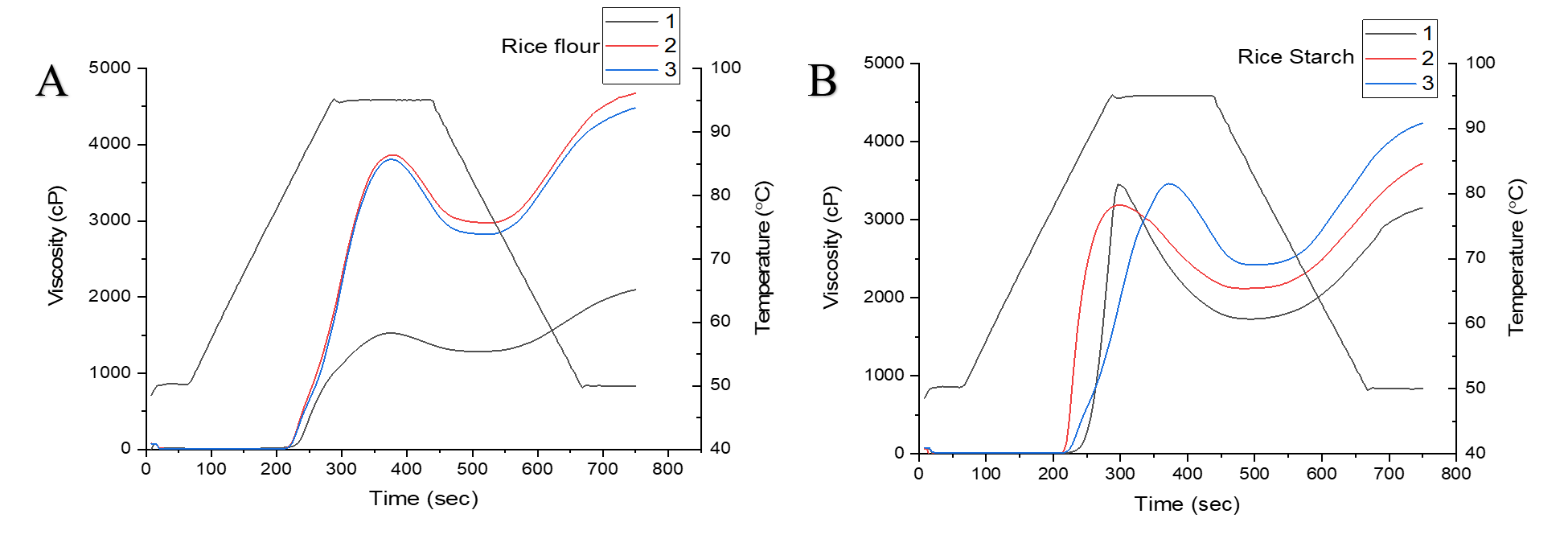
**Fig. S1.**

**Fig. S2.**

**Tables:**

**Table S1. Basic information of samples**

| Sample numbers | Breed name | Grain length (mm) | Grain width (mm) | aspect ratio | Chalkiness grain rate (%) | Chalkiness degree (%) | Husked rice yield (%) | Head rice yield (%) |
| --- | --- | --- | --- | --- | --- | --- | --- | --- |
| 1 | Jinzao-239 | 5.05 | 2.59 | 1.95 | 78.60 | 32.85 | 77.74 | 50.65 |
| 2 | Zhejiang-1702 | 5.14 | 2.63 | 1.95 | 88.50 | 34.65 | 79.48 | 61.75 |
| 3 | Zhongzao-39 | 5.37 | 2.83 | 1.90 | 81.95 | 23.25 | 78.59 | 65.65 |

**Table S2. Sensory scoring rules for rice noodles**

| Level 1 indicators | Secondary indicators | Description |
| --- | --- | --- |
| Odor  15 points | Rice scent  15 points | Rice aroma: 13-15 points |
|  |  | Rice flavor is light: 10-12 points |
|  |  | No rice fragrance, no peculiar smell: 6-9 points |
|  |  | No rice fragrance, peculiar smell: 0-5 points |
| Appearance structure  25 points | Color  6 points | Off-white color peculiar to rice flour: 5-6 points |
|  |  | Normal color, no heterochromia: 3-4 points |
|  |  | Yellowish, black, and heterochromatic color: 0-2 points |
|  | Luster  5 points | Noticeable luster: 4-5 points |
|  |  | Slightly shiny: 2-3 points |
|  |  | Matte: 0-1 points |
|  | Structural integrity  8 points | The structure is compact, the epidermis is not cracked, and it is not easy to break: 7-8 points |
|  |  | A small amount of cracking of the epidermis without breaks: 5-6 points |
|  |  | The epidermis is cracked with powder, easily broken, or drawn: 0-4 points |
|  | Uniformity  6 points | The skin is smooth, and the thickness of the rice flour is uniform: 5-6 points |
|  |  | The skin is smoother, and the rice flour is more uniform: 3-4 points |
|  |  | Epidermal coarseness, rice flour coarseness: 0-2 points |
| palatability  35 points | stickiness  10 points | Smooth and non-sticky to teeth: 8-10 points |
|  |  | Basically, non-sticky to teeth: 5-7 points |
|  |  | Sticky to teeth: 0-4 points |
|  | Hardness & softness  13 points | Moderate hardness and softness: 11-13 points |
|  |  | Slightly soft or slightly hard: 7-10 points |
|  |  | Very soft or very hard: 0-6 points |
|  | Sense of tendon  12 points | Chewy: 10-12 points |
|  |  | Slightly chewy: 7-9 points |
|  |  | No chewiness: 0-6 points |
| Taste  25 points |  | Stronger rice flavor when chewed: 22-25 points |
|  |  | Faint rice flavor when chewing: 18-21 points |
|  |  | No rice flavor, no peculiar smell when chewing: 15-17 points |
|  |  | No rice flavor but peculiar smell when chewing: 0-14 points |

**Table S3. XRD peak analysis of rice starch samples**

| Samples | 1^st^ Peak (Intensity) | 2^nd^ Peak (Intensity) | 3^rd^ Peak (Intensity) | 4^th^ Peak (Intensity) |
| --- | --- | --- | --- | --- |
|  | 2θ Degrees | | | |
| 1 | 15.04 (2018) | 17.24 (2514) | 18.12 (2358) | 23.08 (1971) |
| 2 | 15.75 (1502) | 17.28 (1888) | 18.12 (1810) | 23.02 (1501) |
| 3 | 15.3 (1728) | 17.5 (2099) | 18.04 (1973) | 23 (1582) |
